# Editorial: Who Runs? Psychological, Physiological and Pathophysiological Aspects of Recreational Endurance Athletes

**DOI:** 10.3389/fpsyg.2020.02247

**Published:** 2020-09-11

**Authors:** Pantelis Theodoros Nikolaidis, Beat Knechtle, Alessandro Quartiroli

**Affiliations:** ^1^School of Health and Caring Sciences, University of West Attica, Athens, Greece; ^2^Institute of Primary Care, University of Zurich, Zurich, Switzerland; ^3^Department of Psychology, University of Wisconsin – La Crosse, La Crosse, WI, United States

**Keywords:** aerobic exercise, psychology, physiology, pathophysiology, endurance sport, amateur athletes

Exercise, especially aerobic, has been proven to have many health benefits. Accordingly, the number of individuals engaging in aerobic exercise, especially in endurance-based activities, is continually increasing as evident in the participation trends of endurance running events such as 10 km, half-marathons and marathons. While much is known about the physiological correlates of performance in endurance *elite* runners, little research has been done with regards to psychological, physiological, and pathophysiological aspects of *recreational* endurance runners.

One of the main research questions emerging from observing the increased participation in aerobic exercise and running events centered around the motives of the participants. In recent years, a few studies started exploring the motives leading runners to participate in marathon events, paying special attention to the sex-based and performance-based differences. Unfortunately, these studies based their findings on small samples making their findings to be considered with caution and in need of further research. Another important area of investigation to better understand the participation in endurance exercise and running events focuses on participants' personality traits and how they varied depending on sex, age, performance level, and race distance.

Another important research question related to the increasing participation in aerobic exercise and endurance athletes focuses on the health benefits that aerobic exercise bring to runners. While the long-term benefits of aerobic exercise on physical health have been clearly established in the literature (e.g., reduction of cardiovascular disease), less information is currently available about impact of these activities on participants' psychological health.

Although the health-benefits of exercise has been well-documented, one's participation in aerobic exercise and endurance activities is not without risks. Due to the increasing number of recreational athletes who, without sports experience, engage in endurance activities at much higher intensity than recommended by the international health organizations, the exploration of risk factors on health became of the outmost practical importance. Specifically, it appears imperative to identify and provide evidence-based recommendations for optimal exercise levels to maximize the benefits for physiological as well as psychological well-being, while minimizing possible related risks. The aim of the present Research Topic is to collect manuscripts addressing the existing relationship between participation in endurance-based exercise and psychological and physiological variables across all life-span.

This special issue includes manuscripts focusing on four main areas of research: (a) physiology; (b) psychology; (c) psychology and physiology, and (4) participatory studies. In addition to the original research studies, review articles were also considered.

Among the physiology studies, Mei et al. explored the changes in foot posture, joint kinematics, joint moments and joint contact forces in the lower extremity following a 5 km treadmill run. They found that recreational male runners presented an increased static foot pronation after 5 km treadmill running. They suggested that following mid-distance running foot pronation may be an early indicator of increased lower limb joint loading. Billat et al. sought to establish the relationship between racing strategy, cardiac drift, and performance in recreational marathoner. Runners were grouped into fallers and non-fallers, with the former showing a decrease in speed at the 26th km and a significantly lower performance and higher cardiac drift. The authors found that performance was correlated with the amplitude of the cardiac drift. Based on these findings, they recommend the use of cardiac cost as an objective tool for targeting marathon pace and learning how to self-pace long-distance run.

Nikolaidis et al. examined the relationship of age, body composition, and running speed with muscle strength and flexibility in recreational marathon male runners. The authors reported some age-related differences and showed a moderate relationship between the speed of race and relative isometric strength, but not with the other neuromuscular measures. Due to the relationship between these parameters and health-related physical fitness, the authors encouraged coaches and runners to include stretching and strengthening exercise in their weekly program to enhance all components of health-related physical fitness.

Finally, Olcina et al. described core temperature (Tcore) in top-level and well-trained age group triathletes during the marathon of Ironman World Championship 2014 in Kona-Hawaii under thermal stress conditions. They observed that Tcore increased during the race by ~2°C and correlated negatively with the position in age group (*r* −0.949, *p* = 0.051), i.e., the higher the Tcore, the lower the performance.

Focusing on training, Festa et al. compared the impact of two different training intensity distributions in terms of conditional and performance parameters and time spent training in recreational runners. No differences were found between the polarized endurance training group and the focused endurance training group on any parameter investigated except their total training time. However, athletes in the focused endurance training group obtained similar improvements with a reduced training time. Alvero-Cruz et al. focused their work on exploring the ability to predict athletes' performance of physiological variables obtained in a laboratory as well as in a field test. They observed that half marathon race time could be predicted from the distance ran in the Cooper test as well as from the vVO_2_max values obtained in a laboratory test. Matos et al. analyzed the relationship between variables related to the internal and external loads of training and competition and the perception of well-being in recreational male athletes. Exploring the inter- and intra-week differences, they found that small variations in training stimulus and an increase in maximal oxygen uptake led to improvements in the performance of trail running athletes when considering the running speed in the race.

A second group of the studies in this special issue focused primarily on psychological variable related to running. Waśkiewicz et al. explored marathoners' level of motivation in relation to selected demographics. They reported that experienced and inexperienced marathoners did not differ in their level of motivation, while female and male athletes differ in their motives to compete. These results seem to confirm the existing literature reporting age- and gender-based differences in terms of motivation among marathoners.

Two among these studies aimed to developed psychological profiles of athletes. In the first study, Olmedilla et al. explored the psychological profiles of athletes competing in two cycling sports (triathlon and road cycling). Their aim was to provide an applied model of optimal psychological profiling for sport psychologists, trainers, and coaches to use in promoting their athletes' peak performance. They reported statistically significant differences in few psychological aspects between triathlon and road cyclists and between professionals and amateur athletes. However, they did not find any statistically significant gender-based differences between athletes. In the second study, surveying 42 British athletes, Goddard et al. aimed to explore the construct of mental toughness in extreme ultra-marathoners, while also describing their personality traits. Their results showed a distinct ultra-endurance athlete personality profile characterized by high levels of extraversion and openness to experience. Moreover, their findings also support the literature supporting the argument that mental toughness may be measured by a general personality questionnaire.

On a different line of inquiry, Nogueira et al. sought to explore the relationship between Grit and Dark Traits of personality regarding the appearance of exercise addiction (EA) among amateur endurance athletes. They reported some gender differences, with men scoring higher for addiction levels and for narcissism and psychopathy factors. The authors highlighted how grit may be considered a protective factor against EA, while personality-based factors, such as Machiavellianism, may constitute a risk factor.

Finally, two studies focused their attention on clinically-related topics. For example, Vancini et al. compare quality of life, depression, anxiety symptoms, and profile of mood state of wheelchair basketball and rugby athletes and non-athletes. Not identifying any statistically significant differences between groups, they concluded that wheelchair athletes and non-athletes presented similar level of quality of life, depressive and anxiety symptoms, and mood profiles. Wickström et al. investigated the perception of overuse injury among long-distance runners with different exercise loads. They reported how the overuse injury was perceived to be characterized by the possibility of personal control, treatability, and comprehensibility of the injury context. They also identified some gender-based differences. They concluded that recognition among long-distance runners of the association between their decisions in overuse injury causation is accentuated by increased exercise loads.

In order to bridge the gap between psychologically focused and physiologically focused studies, some of the authors focused their studies on both these aspects. Belinchón-deMiguel et al. explored the possible differences in psychophysiological variables between finishers and non-finishers of a 100-km ultra-endurance mountain athletes. They reported that finishers presented lower levels in psychophysiological parameters before competition compared to non-finishers. They highlighted how their results may be helpful for trainers and athletes to improve their training, and for designing nutritional and psychological interventions aimed to ensure safer participation in these extreme events. Instead Larumbe-Zabala et al. described the longitudinal trends of self-perceptions and their link to physiological performance parameters in recreational marathoners. They reported that improving perceived physical condition predicted self-efficacy, while improvements in motivation were predicted by self-efficacy and perceived physical condition. While no physiological variables appeared to predict changes in psychological variables, their trends over time were correlated. Based on these findings the authors encouraged sport psychologists and coaches to integrate both approaches in training to enhance performance in marathoners. Exel et al. explored the effects of that varying levels of familiarity with the training environment and sensorimotor stimuli originate in the environment have on runners' speed, heart rate regularity degree, and short-term memory. The authors reported how runners showed higher overall short-term memory performance after unusual routes, indicating positive relation to attentional control. Their findings suggest that the contexts of practice may contribute to changing predictability from single to multiple timescales.

Finally, Calogiuri et al. as well as Lepers proposed two participation-based studies. Calogiuri et al. focused their work on mass participation sporting events (MPSE). Specifically, they aimed to analyze trends in women's participation, examined the characteristics of their participation and identified the key factors characterizing women's motivation to participate in different cross-country skiing races. The authors used a mixed methods approach encompassing qualitative analysis of open-ended answers and a machine learning approach to the analysis of sociodemographic characteristics, sport participation, and psychological variables. Their findings corroborate known trends and challenges in MPSE participation, while also providing a novel approach to the study of MPSE.

Lepers investigates the influence of sex on the performance of triathletes in elite and non-elite triathletes. The author reported that, while gender-based difference in triathlon performance may be due to physiological and morphological factors, it is important to explore psychological and participatory factors that may contribute to these differences. He argues that extending the understanding of the factors behind these differences may contribute to promoting more female participation and to helping female triathletes to achieve their maximal performance.

Finally, Gonçalves et al. contributed to this special issue with a review of the literature. They observed that acute and chronic interventions may modify most immune markers, but aspects such as gender, contraceptive pill use in women, physical capacity of the investigated individuals, environment, and type and intensity of the exercises may interfere with these markers as well as the data analysis.

## Author Contributions

All authors listed have made a substantial, direct and intellectual contribution to the work, and approved it for publication.

## Conflict of Interest

The authors declare that the research was conducted in the absence of any commercial or financial relationships that could be construed as a potential conflict of interest.

